# Next generation fatigue crack growth experiments of aerospace materials

**DOI:** 10.1038/s41598-024-63915-x

**Published:** 2024-06-18

**Authors:** Tobias Strohmann, David Melching, Florian Paysan, Eric Dietrich, Guillermo Requena, Eric Breitbarth

**Affiliations:** 1 German Aerospace Center (DLR), Institute of Materials Research, Linder Hoehe, 51147 Cologne, Germany; 2 Metallic Structures and Materials Systems for Aerospace Engineering, RWTH Aachen University, 52062 Aachen, Germany

**Keywords:** Materials science, Engineering

## Abstract

Today’s societal challenges require rapid response and smart materials solutions in almost all technical areas. Driven by these needs, data-driven research has emerged as an enabler for faster innovation cycles. In fields such as chemistry, materials science and life sciences, automatic and even autonomous data generation and processing is already accelerating knowledge discovery. In contrast, in experimental mechanics, complex investigations like studying fatigue crack growth in structural materials have traditionally adhered to standardized procedures with limited adoption of the digital transformation. In this work, we present a novel infrastructure for data-centric experimental mechanics in the field of fatigue crack growth. Our methodology incorporates a robust code base that complements a multi-scale digital image correlation and robot-assisted test rig. Using this approach, the information-to-cost ratio of fatigue crack growth experiments in aerospace materials is significantly higher compared to traditional experiments. Thus, serves as a catalyst for discovering new scientific knowledge in the field of structural materials and structures.

## Introduction

Today’s societal challenges require rapid response and smart materials solutions in almost all technical areas. Motivated by these needs, data-driven research has emerged as a new paradigm to enable faster innovation cycles^1,2^. In this context, automatic and even autonomous laboratories which generate and process data are being developed to accelerate knowledge discovery in many fields, such as materials science, chemistry, and life sciences^1–14^. In contrast, the number of studies investigating similar concepts for mechanical testing^15^ is very limited and especially complex experiments such as fatigue crack growth (FCG) in structural materials have historically followed highly standardized procedures^16^ with limited digitalization. These experiments are fundamental to understand the process-microstructure-property relationship in a wide range of applications where fatigue cracks are inherent to structural design, e.g. in aircraft structures^17^.

In conventional FCG experiments, the stress intensity factor (SIF) is calculated as a function of specimen geometry, load, and crack length using analytical formulas or modelling tools such as finite element analysis. The resulting d*a*/d*N* − Δ*K* curves (where d*a* is the incremental crack length difference per load cycle *N* and Δ*K* is the respective cyclic SIF) are suitable for lifetime estimations, but do not provide information on physical (or local) crack propagation mechanisms. Moreover, the analysis of such experimental data usually involves manual steps and requires highly qualified domain experts. Consequently, the information-to-cost ratio is notably low.

To increase this ratio, research shows promising advances e. g. using synchrotron X-rays for diffraction based approaches^18^ or computed tomography complemented by digital volume correlation^19^. However, for the everyday lab operation, test equipment must be achievable and easy to implement. As a consequence, for fracture mechanics, digital image correlation (DIC) has become a state-of-the-art method for generating full-field information of displacements and strains during crack growth experiments^20–42^. For instance, DIC has been successfully applied together with numerical or analytical approaches to calculate the J-integral, stress intensity factors or T-stress^20,23,25–27,29,31–34,37–41^, analyse the crack tip plastic zone^21,22^, crack opening displacements^36,37^, or mechanisms acting locally at the crack tip, such as strain accumulation^24^. However, the non-automated acquisition and analysis of DIC data is not readily scalable in terms of time and the amount of information that can be processed. A key requirement for automation is the rapid and robust detection of the fatigue crack path and, even more important, the crack tip. To this purpose, artificial neural networks have recently been trained to reliably and fully automatically detect fatigue cracks in DIC data^43,44^. Nevertheless, automated DIC remains very challenging, particularly for cases in which variable regions of interest much smaller than the specimen are studied, as it is the case of high resolution DIC (HRDIC). To address this challenge, Paysan et al. recently developed algorithms for a robot-assisted test rig that can be used to automatically scan a specimen surface during fatigue crack growth experiments using an optical microscope for HRDIC analysis^45^.

Overall, literature shows that the integration of experimental DIC (especially HRDIC) and algorithmic analyses yields deep understanding of crack growth behaviour and its underlying mechanisms^40^. However, this approach requires entirely new test infrastructures, including interconnected hardware, i.e. the DIC system/s, the robotic systems and the test rig, as well as a robust code base for data management that include data acquisition, analysis, and storage. In addition, it is important to ensure that the generated data meets the criteria of being findable, accessible, interoperable, and reusable (F.A.I.R.) so that it can be utilized for data-driven research in a sustainable manner^46^.

In this work we present the digital backbone complementing our novel test infrastructure for data-centric fatigue crack growth experiments. Our methodology includes a fracture mechanics code base published as a Python library called “CrackPy—Crack Growth Analysis in Python” which complements our multiscale DIC and robotic-assisted test rig. We demonstrate the effectiveness of our experimental setup by comparing it to conventional experiments. Our results show that the integration of experimental mechanics with robotic systems and digital tools enables automation as well as deeper insights into materials behaviour and, as a result, the information-to-cost ratio is increased.

## Methodology

### Infrastructure for next generation fatigue crack growth experiments

**Figure 1:**
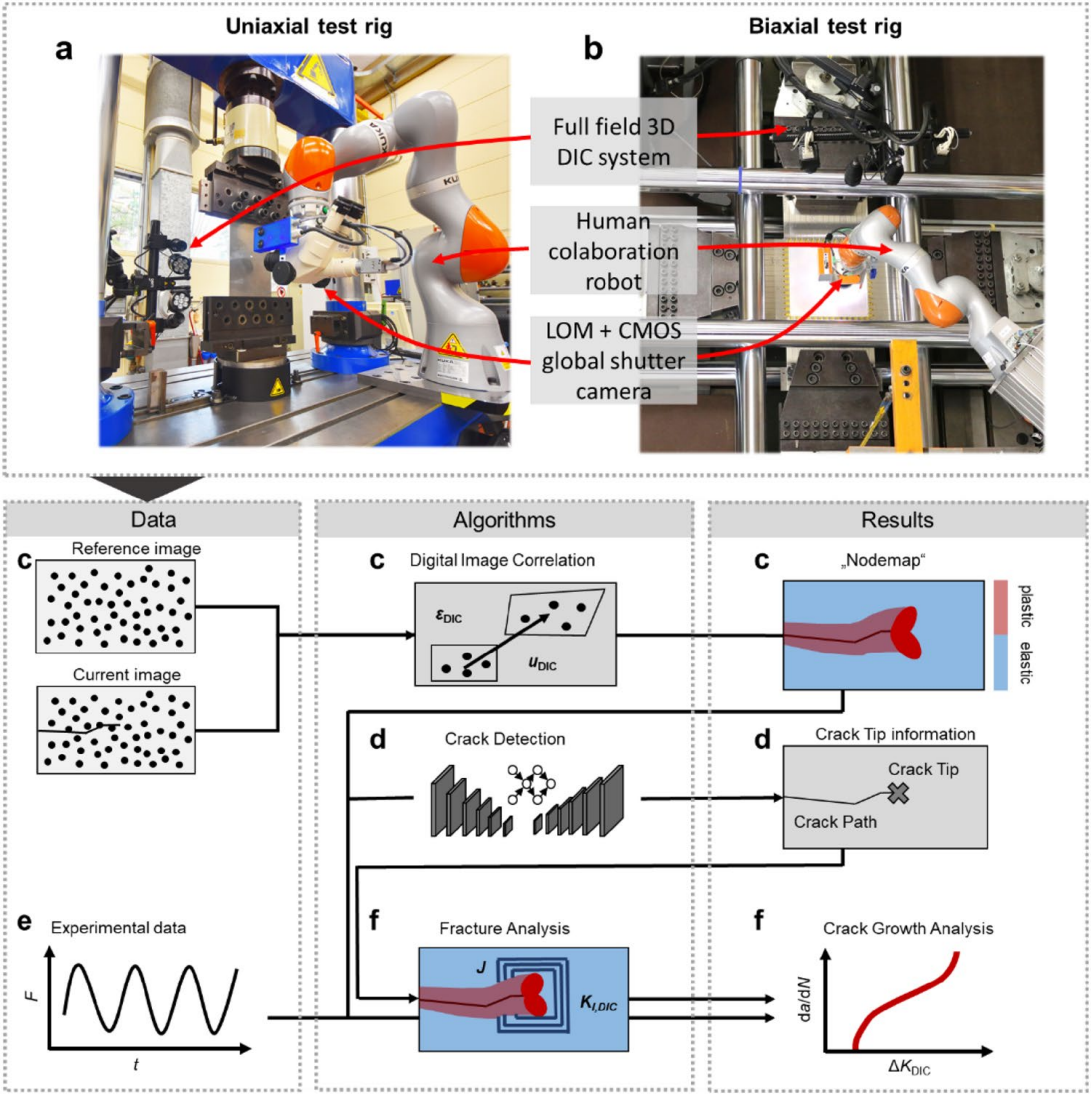
Overview of data flow from acquisition to data analysis, integrating raw data with algorithms for results processing and analysis. (**a**, **b**) The uniaxial and biaxial test setups, respectively. We integrate a full field commercial DIC system, and a robot carrying a light optical microscope (LOM) equipped with a CMOS global shutter camera. (**c**–**f**) gives an overview of the process showing flow of data and the use of algorithms producing results. F is the applied load, t, the time, ${u}_{DIC}$ and ${\varepsilon }_{DIC}$, the displacements and strains calculated by DIC.

Figure 1 provides an overview of the (digital) infrastructure developed showing the flow of information between hardware and algorithms producing (raw) data and results. Starting with a commercial 3D DIC system (see Supplementary material “detailed_methodology_description.pdf” for full details), full-field displacements and strains are calculated on the specimen’s surface (Fig. 1c). At each time step during the experiments, the DIC data is saved in a node-wise neutral (.txt) format—we call it the “nodemap” file. Then, the current crack tip position and crack path location are detected based on the analysis of the DIC displacement field. To this purpose, we use trained convolutional neural networks (CNNs) (see Fig. 1d) as explained in detail in our previous works^43,44^. The CNN models were trained using supervised learning on a data set containing DIC displacement fields, manually labelled with the crack path and crack tip position^47^. The network focusses its attention on the characteristic crack tip field ahead of the crack to accurately detect its position^44^. The crack detection can be carried out in situ during the fatigue crack growth experiment to feed the crack tip information to the DIC system, the robot, and the test rig controller enabling a closed-loop experiment, or ex situ for all acquired time steps. A second DIC system is carried by a cobot using a light optical microscope (LOM) for higher magnification of the displacements and strains. This second system can therefore be used to perform HRDIC by moving the microscope to a region of interest using the crack tip information or by scanning the entire specimen’s surface in a checker board pattern (if the specimen is small). To ensure that the region of interest appears sharply in the focus of the microscope, the robot’s position can be fine-adjusted fully automated according to the implementation of Paysan et al.^45^. The hardware is fully automated for uniaxial test rigs (Fig. 1a)^45^ and was used in this setup to obtain the data discussed in this study. Moreover, the whole system has recently been adapted for a large biaxial test rig (Fig. 1b, see also Supplementary Video 1).

The data analysis provides several fracture mechanical parameters such as SIFs, T-stress or higher order terms of the Williams series based on fitting methods or integral techniques (Fig. 1f). In particular, the utilization of Williams series coefficients condenses the crack tip field into a concise feature vector. This approach enables data-driven evaluation by representing the essential characteristics of the crack tip near-field in a lower dimensional space. These functionalities are implemented in *CrackPy*^48^ and described in detail in the next section. The goal is to generate comprehensive datasets according to F.A.I.R. principles for each experiment.

### CrackPy

**Figure 2:**
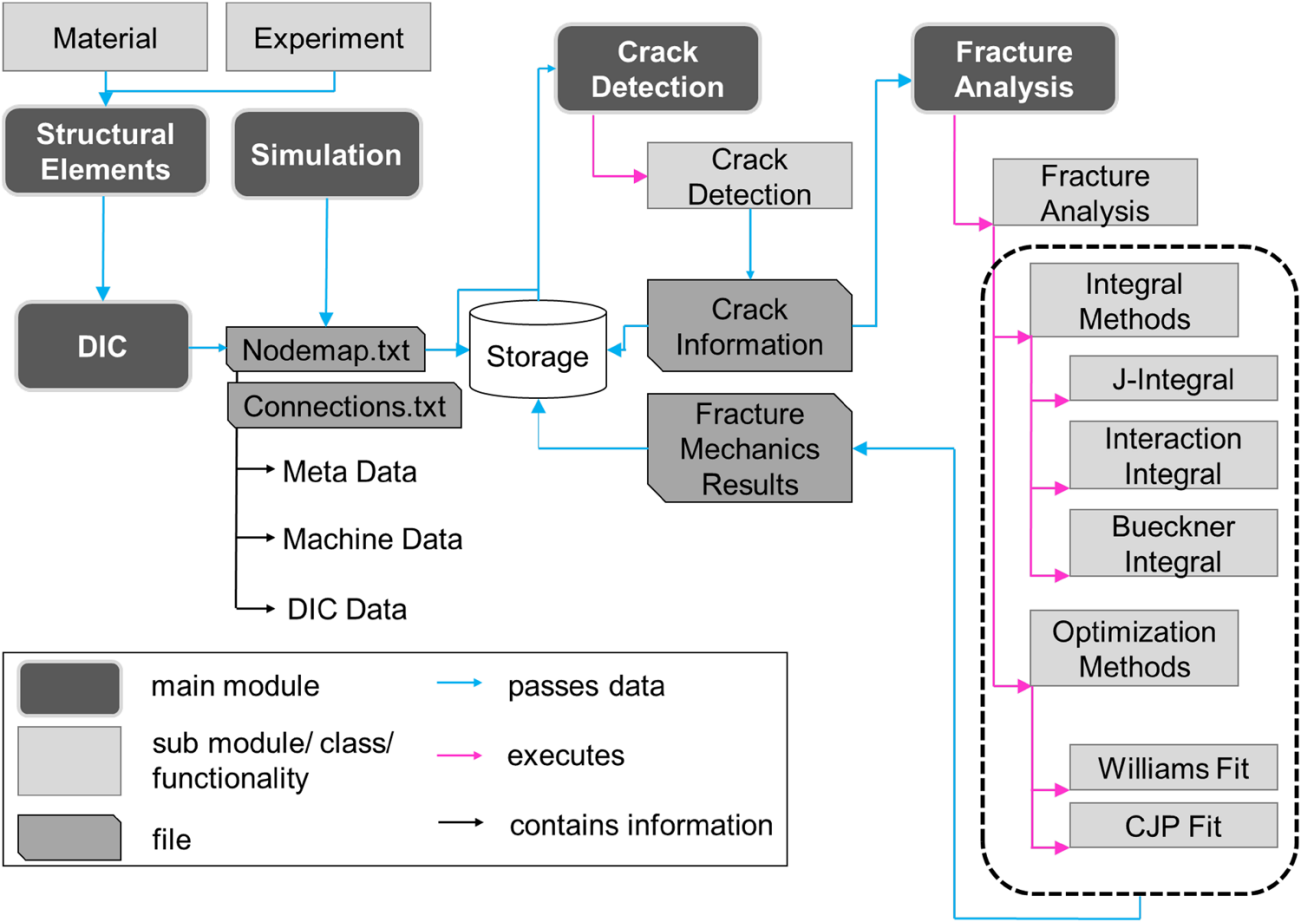
Structure of Python-based library *CrackPy, v.1.1.1 *^48^*.*

We developed a Python-based library called *CrackPy*^48^ to automate the data analysis pipeline. The library is structured according to Fig. 2. A *Structural Element* module provides classes that contain metadata, for instance the *Material* class. The *Material* class contains information about the material’s physical parameters, like Young’s modulus, shear modulus, stiffness matrix, etc. As mentioned above, the nodal coordinates, displacement vectors and surface total mechanical strain tensors are stored in a neutral structure as text files (“*Nodemap*”) together with corresponding metadata. The metadata contain the experiment name, the DIC parameters, the specimen and material investigated, and information on the actual time or load step. A “*_connection.txt*” file stores the mesh information of the DIC evaluation domain. The specific file structures can be created from any file type or system that provides node-wise results data. For instance, it can be generated from *DIC or simulated* (e.g. finite element) data. In our case, we used DIC data obtained from a commercial DIC system. The "nodemap" and "connection" files can be used to visualize the data or to save all the information in ".vtk" (Visualization Toolkit) file format. Once the nodal DIC data are stored in this “Nodemap” format, the actual crack analysis can begin. Crack information such as the crack angle and crack tip position can be detected automatically using the *Crack Detection* module based on trained convolutional neural networks^43,44^ or set manually (e.g. in case of simulations, where the crack information is known a-priori). The network architectures together with the weights of the trained CNN models are available in Ref.^49^. The crack tip information is then stored in a file “*Crack Information”* (see Fig. 2) and is used as input for the *Fracture Analysis* module. The analysis is carried out using surface information of the specimen. Consequently, a homogeneous crack throughout the thickness visible on the surface is assumed together with a sufficient length of the crack with respect to the specimen size and field of view of the DIC system. *CrackPy* features a wide range of methods and algorithms. Currently (*CrackPy 1.1.1*), the following methods are implemented:

1. Calculation of the energy release rate during crack propagation by the *J integral*^20,50^
2. Calculation of stress intensity factors (mode I, mode II) using the *interaction integral technique*^51,52^
3. Calculation of *higher order singular terms (HOSTs)* or *higher order regular terms (HORTs)* of the Williams series^53^ (including T-stress) using *Bueckner's conjugate work integral *^54^ together with the interaction technique described in Refs.^55,56^
4. Calculation of *higher order singular terms (HOSTs)* or *higher order regular terms (HORTs)* of the Williams series^53^ (including *K*_I_, *K*_II_ and T-stress) by fitting the theoretical displacement field to the experimentally measured (or simulated) data^57^
5. Calculation of stress intensity factors that take into account plasticity effects by fitting the theoretical displacement field of the *CJP model*^58^ to the experimentally measured (or simulated) data^59^

Methods 3–5 are receiving increasing attention: The methods describe the whole crack tip field in an alternative way considering higher-order terms of the Williams series and plastic effects. While the fitting methods (4 and 5) rely solely on displacements, the integral methods (1–3) also depend on stresses. Since DIC can only provide displacement and strain measurements, the stress fields must be calculated using an appropriate material model. For *CrackPy*, we use a linear-elastic material law—a good approximation in the absence of plastic deformation—and choose an integration domain away from the plastic zone surrounding the crack. The result of the analysis is then stored in *Fracture Mechanics Results* as structured text files and plots. The large amount of stored data, in the long-term, enables data-centric analyses, including techniques such as clustering, machine learning, and symbolic regression^60^. Such techniques need data to uncover patterns, make predictions, or build new physical models^46^.

## Results

**Figure 3:**
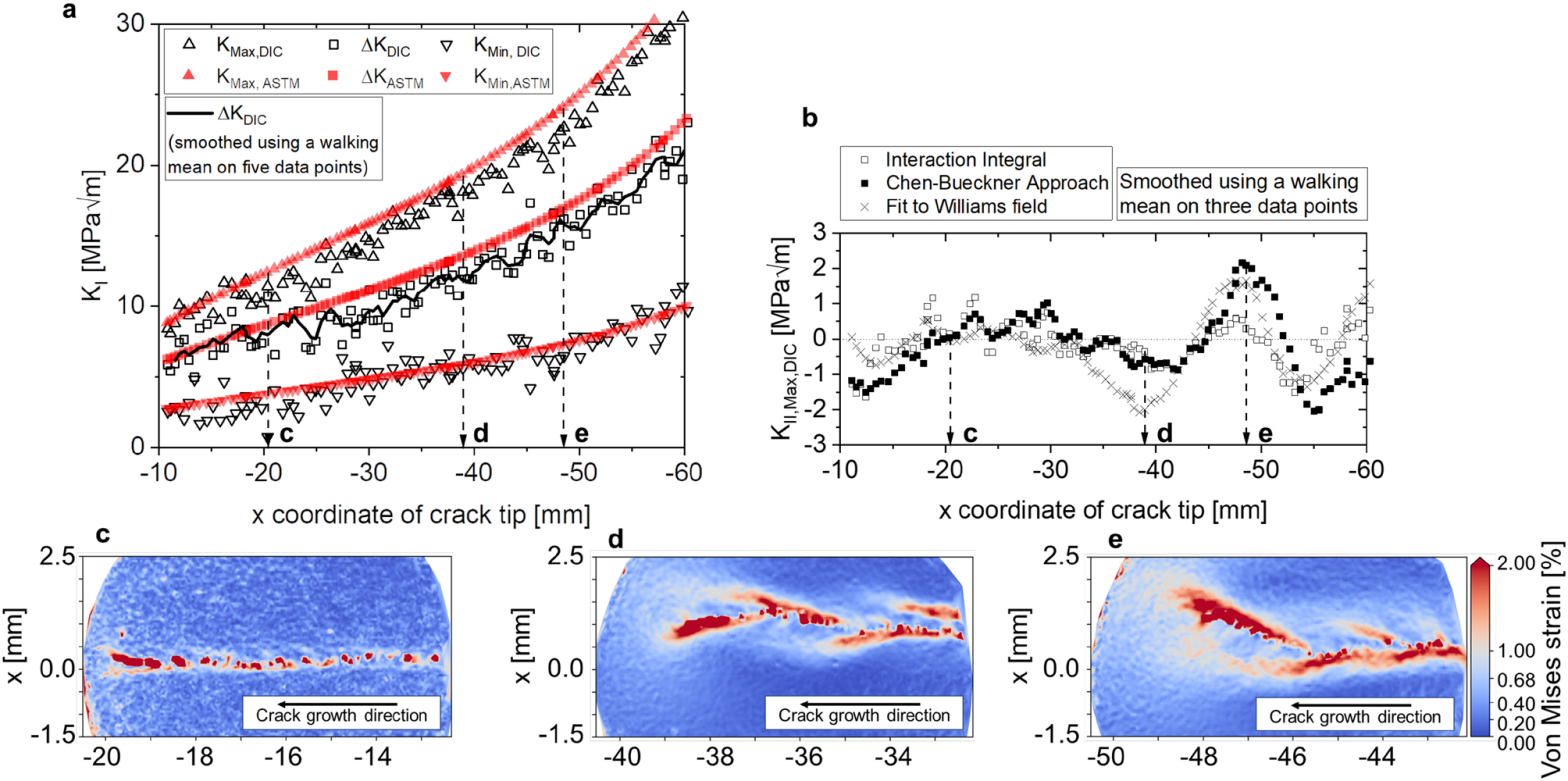
Comparison of K_I_ (**a**) and K_II_ (**b**), calculated conventionally following ASTM E647 (red) and using the interaction integral on DIC data (black). The data was smoothed via walking mean using a 5-data-points (K_I_) and 3-data-points (K_II_) window size. (**c**) The local von Mises strain field for a relatively straight crack at crack length a = 20 mm. (**d**, **e**) A branched crack at higher crack lengths of ~ 38 and ~ 48 mm, respectively. For DIC data at multiple time steps, the reader is referred to *Supplementary Video 2*.

**Figure 4:**
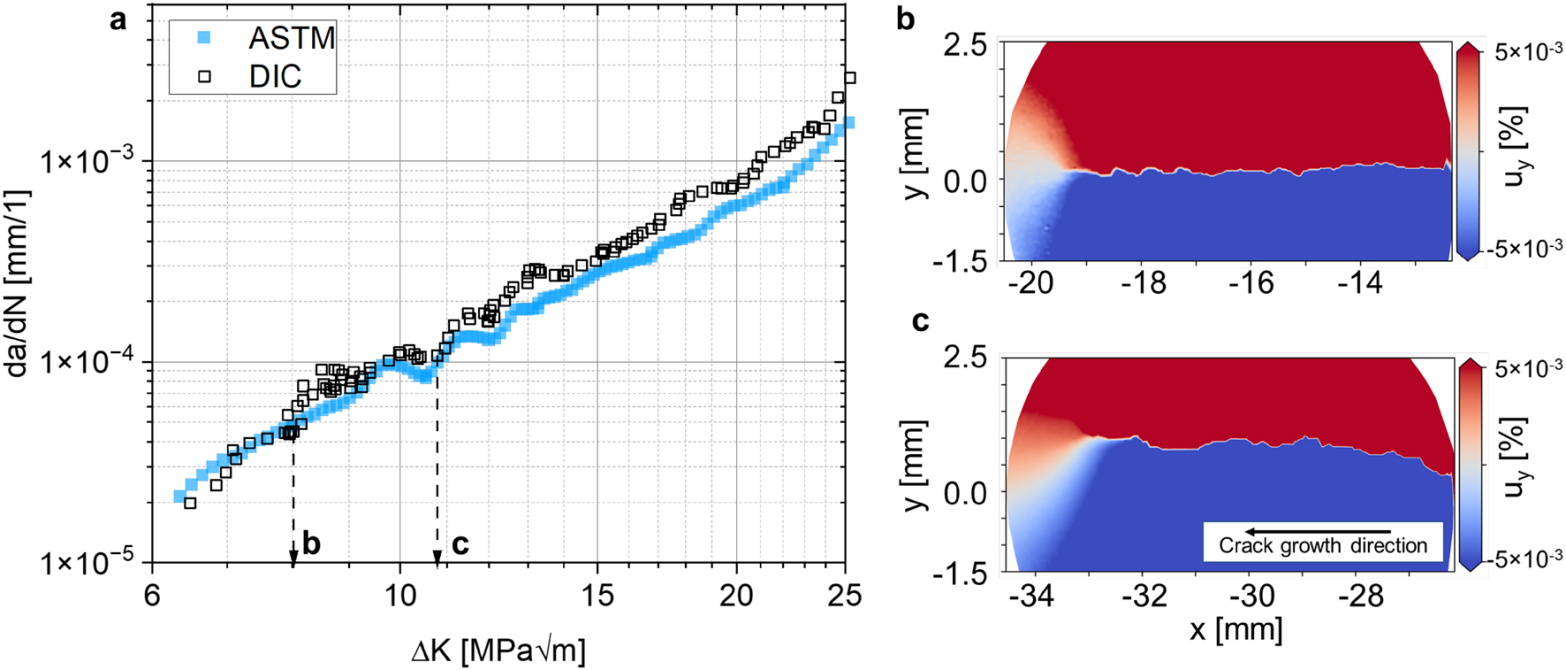
FCG analysis of AA2024-T3 following the conventional ASTM methodology (a, blue) and DIC-based (**a**, black) with the interaction integral, both smoothed via a walking mean with seven data points window size. Both curves are close together, however, the conventional method only yields integral results, i.e. a − N and da/dN − ΔK. In contrast, we can now reveal local effects by high resolution DIC. (**b**) The local displacement field around the crack at low ΔK, with a mostly straight crack path and, (**c**) a branched crack path at higher ΔK resulting in non-symmetric displacements in front of the crack tip.

Figure 3a shows the mode I SIF at minimum and maximum load as well as the cyclic mode I SIF as a function of the x coordinate of the crack tip for a cold rolled AA2024-T3 aluminum alloy tested in L–T orientation. Figure 4a presents the same data plotted as d*a*/d*N* vs. Δ*K*. For this experiment, we integrated our robot-based infrastructure into a servo-hydraulic uniaxial test rig^45^. The whole system is shown in Fig. 1a. We applied a sinusoidal cyclic load at 20 Hz ranging from *F*_min_ = 4.5 kN to *F*_max_ = 15 kN, i.e. *R* = *F*_min_/*F*_max_ = 0.3 on a middle tension specimen of width *W* = 160 mm and thickness *t* = 2 mm. In depth details of the experimental conditions are specified in the supplementary material “detailed_methodology_description.pdf”.

We compare the conventional analysis, i.e. using ASTM E647^16^

$$\Delta {\text{K}}_{{{\text{ASTM}}}} = \Delta {\text{K}}_{{{\text{I}},{\text{ASTM}}}} = \frac{{\Delta {\text{F}}}}{{\text{t}}} \cdot \sqrt {\frac{\pi \alpha }{{2{\text{W}}\cos (0.5\pi \alpha )}}} ,$$

where α = 2*a*/*W*, with the DIC-based results calculated using the interaction integral^48,51,52^ for images captured at minimum and maximum load throughout the experiment. The conventional analysis assumes a symmetric crack growth. Thus, the overall crack length can be estimated by direct current potential drop (DCPD), i.e. *a*_x_ = 2*a*/2. One advantage of the DIC-based method is that both sides of the crack can be analyzed individually. Here, we show only one side of the crack—referred to as “left” side, i.e. the crack growths along the negative x direction with respect to the coordinate system located in the specimen center—and detected the actual crack tip position using our trained CNN. Overall, the novel method yields similar results to the conventional one in terms of *K*-*a* and d*a*/d*N* − Δ*K* (Figs. 3a and 4a, respectively). The curves are well aligned for small Δ*K* = 7.0–9.5 MPa√m. In contrast, the curves from the two methods seem to be shifted away from each other for Δ*K* > 9.5 MPa√m. This effect is due to the difference between the conventional and the DIC-based methods in terms of calculated SIFs (since d*a*/d*N* is almost identical for both methods). In contrast to conventional methods, the continuous access to the DIC and HRDIC data enables now a detailed analysis of such effects: Figs. 3c–e and 4b,c show the von Mises equivalent strains and the vertical (y) displacement around the crack tip and crack path obtained by HRDIC for representative crack growth states during the experiment, respectively. While the crack path is mostly straight at low Δ*K* (Fig. 3c), a tortuous crack path propagates later (Fig. 3d,e). From a fracture mechanics perspective, it can be inferred that this zig-zag-like crack path may be a consequence of secondary cracks that result in a reduction of the effective stress intensity at the primary crack tip^61^. This has a large effect on the SIF at maximum load but a smaller one at minimum load. Consequently, the effective cyclic SIF (based on DIC results) is lower and more realistic than that obtained using the conventional method based on the ASTM that assumes a fully straight crack. The effect is well aligned with the evolution of *K*_II_ throughout the experiment: Conventionally, *K*_II_ is considered to be zero using the ASTM method because the crack path is assumed to be perfectly straight, and thus, a pure mode I state is assumed. However, we find that *K*_II,DIC_ ranges from − 2 to 2 MPa√m (Fig. 3b) as soon as crack branching begins (Figs. 3d,e, 4c). These effects are captured locally and continuously throughout the experiment by our method because the results are a consequence of the actual displacements and strains. We show more data of several time steps in Supplementary Video 2. In contrast, the conventional method is unable to detect such phenomena because the SIFs are calculated based only on crack length, load and specimen geometry. Furthermore, we compare results for *K*_II_ based on three different approaches computed simultaneously in *CrackPy*: the interaction integral^51,52^, the Bueckner integral^55^ and a fit of the theoretical displacements to the experimental data with respect to Williams’ formulation^53^ using the Levenberg–Marquardt algorithm^62^. Although the three methods yield quantitatively different values for *K*_II_, i.e. the interaction integral underestimates *K*_II_ compared to the other two methods, the overall trend is similar for all of them.

**Figure 5:**
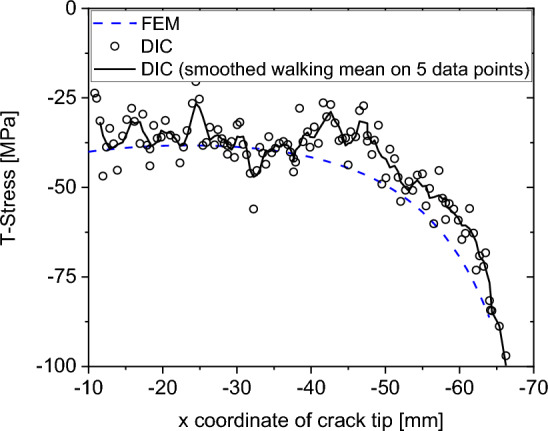
T-Stress as a function of crack tip coordinate x (left side of the crack) for the experimental DIC data (using the interaction integral) and the theoretical FEM solution.

Fig. 5 shows the evolution of the T-stress as a function of the crack length (in terms of the crack tip’s x coordinate). The T-stress acts parallel to the crack and is associated to the first non-singular term of the Williams series expansion. In literature, it is usually correlated to crack path stability^63^. Here, we determine the T-stress using the experimental DIC data and the interaction integral method and compare it with the theoretical finite element method (FEM) solution. The finite element model considers a linear-elastic constitutive law (E = 72 GPa, ν = 0.33) and a structured 2D plane element mesh with an element size of 0.04 × 0.04 mm^2^. Again, two regions can be identified: a first region between − 20 mm > x > − 38 mm where both results are close and the DIC results are scattered around the FEM solution. Beyond − 38 mm, the DIC results are higher than the FEM solution. We associate this characteristic with the transition from an almost straight crack path in the first region, to a more tortuous and branched crack path in the second region (see Fig. 3c–e, respectively).

We have shown the first and second term of the Williams expansion, i.e. K_I_, K_II_, and T-stress. Moreover, as described in the section “CrackPy”, it is possible to calculate higher-order terms of the Williams expansion using Bueckner’s conjugated work integral or by fitting the theoretical displacement field to the experimental data. There is no evidence in the literature about the physical meaning of these higher-order terms although some studies show an effect on crack growth: Higher-order terms can be necessary to match the crack tip near field with the remote geometry or boundary conditions^64^. Moreover, at least theoretically, the third regular term is responsible for crack propagation stability^65^. We refer to Ref.^56^ for a parameter study of higher-order terms carried out on FE simulations of different standard specimen geometries. A systematic analysis of these higher-order terms, especially for experimental DIC data, will enable new perspectives to investigate their influence on e.g. fracture modes, crack path stability or crack branching. Furthermore, higher order terms can be used to condense the complexity of the crack tip field into a discrete feature vector. This feature vector allows a data-centric approach while it also enables a complete reconstruction of the crack tip field. Exemplary, we show terms A_1_–A_4_ and B_1_–B_4_ for the presented experiment in the Supplementary material (Supplementary Fig. 1) by fitting the theoretical displacements to the experimental ones.

## Discussion

With our digital backbone, i.e. CrackPy complementing our DIC- and robot-based test infrastructure, we have continuous access to displacement and strain data throughout the experiment and at two different length scales, i.e. global 3D DIC and local 2D HRDIC. This alone is a tremendous increase of the information-to-cost ratio for FCG experiments because local effects on the specimen surface can be captured for any number of time and load steps. This opens up the possibility of analyzing intrinsic and extrinsic crack growth effects, individually. Table 1 shows a comparison of the conventional and the robot-assisted DIC-based methods for fatigue crack growth experiments on eight criteria.

**Table 1:** Comparison of the conventional fatigue crack growth testing and our new methodology.

	Conventional FCG experiments	Robot-assisted FCG experiments
*a*-*N*	The direct current potential drop (DCPD) technique provides integral values assuming a straight crack and symmetrical crack growth	Both sides of the crack can be analyzed individually; the actual crack path is detected during the experiment. Applicable also for non-straight crack geometries
d*a*/d*N*-Δ*K*	Straight crack is assumed, i.e. *K*_II_ = 0 with idealized loading conditions	Calculation of *K* based on experimental DIC-measured displacement and strain field reflecting actual loading conditions and crack deflection
T-stress	Can be calculated using analytical formulations or finite element analysis. Necessitates idealized assumptions	Calculated based on experimental DIC data
Higher-order terms	Can be calculated using finite element analysis assuming idealized test conditions and material properties	Calculated based on experimental DIC data
Local effects (e.g. crack deflection, crack branching/bifurcation, crack closure)	Captured as integral effects on the crack growth rate; a local analysis is only possible by post-mortem fractography	Captured in situ locally using high resolution DIC; can be combined with traditional methods like fractography post-mortem
Specimen geometry	Standard geometry	Arbitrary geometry
Duration of one experiment	~ 1–2 days, depending on frequency, material and specimen size; This time usually involves few or no manual steps	~ 5–6 days; This time usually involves few or no manual steps
Data set size for one experiment	~ KBs to MBs	> 100 GB

Both methods are capable to generate *a*-*N* and d*a*/d*N*-Δ*K* data. However, the robot-assisted DIC-based methodology has several further advantages: First, the DIC images can be used to calculate fracture mechanics parameters such as SIFs, T-stress, etc. using integral techniques based on actual experimental data rather than analytically, which permits eliminating assumptions. Apart from SIFs, higher-order terms of the Williams expansion are conventionally calculated using FEM, i.e. rely on an idealized material, geometry, etc., neglecting e.g. microstructural effects. By means of Chen’s approach using the conjugated work integral^55^, it is possible to calculate these parameters based on the experimental DIC data. In addition, the use of full-field data increases confidence in experimental results. Moreover, redundancy is achieved through multiple data sources, overlapping data and independent evaluation algorithms. This comprehensive approach promotes data reliability and facilitates the differentiation between scatter, anomalies and true effects.

The overall time needed to run one single experiment strongly depends on the investigated material, load conditions, specimen size and environmental conditions. For our experiments, the time needed increased from 1–2 days using the conventional ASTM method to ~ 1 week with the new test infrastructure. In both cases, the test procedure is almost completely automatized and we believe that the additional time needed is compensated by the much larger outcome from the experiment.

A notable feature of the methodology introduced in this work is the large amount of data acquired per experiment (> 100 GB). This requires a significant increase of data storage capabilities compared to conventional testing methods. Handling such large amounts of data requires a storage strategy in line with the principals of findable, accessible, interoperable, and reusable data^66,67^. The 3D DIC data shown here is publicly available on Zenodo, with a digital object identifier (DOI)^68^ serving as persistent and unique identifier. Thus, the data is stored for open, long-term access and is *findable*. The data and metadata can be accessed via a web browser or the Zenodo REST application programming interface (API)^69^. We distinguish between metadata at different levels. More precisely, we store metadata describing the whole experiment, the material with its manufacturing process separated from the data^68^ *accessible* and *interoperable* even if the data was no longer available^66^. On the other hand, we store metadata describing a respective time step directly within the data files to minimize the risk of mixing up metadata or data. To this purpose, we designed the “nodemap”-file as a specific structure of text file including a long header containing all relevant metadata as keyword value pairs. We describe the experiments accurately on the highest level of metadata meeting fracture mechanics community standards. In addition, we explain the vocabulary of the metadata using dictionaries to avoid confusion of any terms. We believe that these accurate descriptions make it easier for humans to understand the origin of the data, while it remains hard for machines to understand the metadata fully automatically. Thus, it becomes more and more important to design standards describing experiments, material and processes of the domain of experimental mechanics for the purpose of machine interoperability, and therefore, *reusable* data.

## Conclusions

In summary, we developed, implemented, and showcased the digital backbone for a new generation test infrastructure for complex crack growth experiments. Therefore, we complemented DIC with robotics and a robust fracture mechanics code base. The novel methodology increases the information-to-cost ratio for one experiment tremendously making local effects accessible throughout the experiment. Data are obtained and stored according to F.A.I.R principals. As a consequence, results become more usable, understandable and reliable.

### Supplementary Information

Supplementary Information 1.

Supplementary Information 2.

Supplementary Video 1.

Supplementary Video 2.

## Data Availability

The research data are publicly available as Zenodo dataset^68^.

## Code availability

The code to reproduce results, i.e. *CrackPy (version 1.1.1)* is available on github https://github.com/dlr-wf/crackpy^48^ under MIT licence. However, the authors recently released an updated version.
