# Supplementary material for: Next generation fatigue crack growth experiments of aerospace materials
Source: Sci Rep. 2024 Jun 18;14:14075. doi: 10.1038/s41598-024-63915-x (PMC11189514; doi:10.1038/s41598-024-63915-x)
Supplement: Supplementary file 1 — Supplementary Information 1. [file 41598_2024_63915_MOESM1_ESM.pdf]

Supplementary material for article:

Next generation fatigue crack growth experiments of aerospace materials

Tobias STROHMANN[1], David MELCHING[1], Florian PAYSAN[1], Eric DIETRICH[1],

Guillermo REQUENA[1, 2], Eric BREITBARTH[1]

[1] German Aerospace Center (DLR), Institute of Materials Research, Linder Hoehe, 51147 Cologne, Germany

[2] Metallic Structures and Materials Systems for Aerospace Engineering, RWTH Aachen University, Aachen 52062, Germany

1 Detailed description of experimental methodology

1.1 Digital image correlation

We acquired data using the commercial 3D DIC System GOM Aramis 12M and the software GOM Aramis Professional 2021. We supported the commercial system with *CrackPy* to store metadata when images are captured, to control the experiment, i.e. by on-the-fly crack detection, and to store the results as human-readable text files that combine metadata and facet results in terms of displacements and strains. We used a facet size of 20×20 pixels with a 16 pixels facet distance. One facet covered an area of 0.614×0.614 mm². An exposure time of 3000 ms was used.

1.2 Robot-assisted high-resolution digital image correlation

We used a KUKA LBR iiwa cobot which carries a Zeiss STEMI 206C light optical microscope equipped with a Basler a2A5320-23µmPRO global shutter CMOS camera to take high resolution images. The test rig is explained in detail by Paysan et al. in [1]. The chosen field of view was 10.2×5.7 mm² which was covered by facets with a size of 40×40 pixels and a facet distance of 30 pixels corresponding to a spatial resolution (distance between two facet center points) of 0.05 mm. An exposure time of 2000 ms was used.

1.3 Crack detection

The detection of crack paths and crack tips can be performed *in situ* or *ex situ* using the *Crack Detection* module of *CrackPy* [2] on any number of load steps. Therefore, we used two trained convolutional neural networks, i.e., the so-called *ParallelNets* to find the crack tip and the so-called *UNetPath* to detect the crack path. Network architectures can be found in *CrackPy*. For the crack detection, we use the global 3D DIC displacement fields as input data. The crack angle is approximated by fitting a line through the detected crack path near the tip. Moreover, the crack tip detection model is explainable and can be monitored using attention visualizations based on Grad-CAM [3]. For further details we refer to [4, 5].

1.4 Experimental procedure

1.4.1 Material

A commercially available AA2024-T3 aluminium alloy was used for the FCG experiments. This alloy was chosen because it represents one of the most relevant alloys used in the aircraft industry, particularly for fuselage structures. The material was tested in L-T orientation, i.e. rolling direction (L, elongated grains) parallel to the load axis and transverse direction (T) perpendicular to the load axis. A middle tension (MT) specimen with a thickness of 2.0 mm and a width of 160 mm was cut from a rolled sheet. The material has a Young's modulus E = 72.0 GPa and poisson's ratio μ = 0.3.

1.4.2 Fatigue crack growth

We used a standard uniaxial test rig. We applied a cyclic load ranging from F_{min} = 4.5 kN to F_{max} = 15 kN, i.e. R = F_{min}/F_{max} = 0.3 on a MT specimen (width W = 160 mm, thickness t = 2 mm). We measured the crack length by direct current potential drop (DCPD) following ASTM E-647 [6] to control the experiment. However, when we analyzed the experiment, we used DCPD for the conventional method (ASTM) and used the crack tip achieved from the trained neural network (see section 1.3) for the novel method.

1.4.3 Image acquisition during fatigue crack growth

We acquired reference images for the DIC calculations before the experiment. For the global DIC, this is simply an image of the unloaded specimen. For the local HRDIC the reference images are acquired in a checker board pattern with an overlap of 70 % covering the whole specimen surface. The depth of focus is calibrated for each image and the camera is aligned to the specimen's surface according to Paysan et al. [1]. The spatial position of the robot (and consequently of the microscope) are saved as metadata for each of these reference images. The test was stopped every 0.5 mm of crack extension. Then, the crack length was used to determine the region of interest around the crack tip and the robot moved to the corresponding reference images' positions to acquire new images at the current loading conditions. Therefore, the region of interest was defined as a square covering the crack tip region of 18 × 9.2 mm². This was done for F_{max}, F_{min}, and F_{min} + 0.5 (F_{max}-F_{min}).

2 References

1. Paysan F, Dietrich E, Breitbarth E (2023) A robot-assisted microscopy system for digital image correlation in fatigue crack growth testing. Experimental Mechanics. https://doi.org/10.1007/s11340-023-00964-9
2. Strohmann T, Melching D, Paysan F et al. (2022) Crack Analysis Tool in Python - CrackPy. Zenodo
3. Selvaraju RR, Cogswell M, Das A et al. (2020) Grad-CAM: Visual Explanations from Deep Networks via Gradient-Based Localization. Int J Comput Vis 128:336–359. https://doi.org/10.1007/s11263-019-01228-7

4. Strohmann T, Starostin‐Penner D, Breitbarth E et al. (2021) Automatic detection of fatigue crack paths using digital image correlation and convolutional neural networks. Fatigue Fract Eng Mater Struct 44:1336‐1348. https://doi.org/10.1111/ffe.13433
5. Melching D, Strohmann T, Requena G et al. (2022) Explainable machine learning for precise fatigue crack tip detection. Sci Rep 12:9513. https://doi.org/10.1038/s41598-022-13275-1
6. ASTM International ASTM E-647-13: Standard Test Method for Measurement of Fatigue Crack Growth Rates:684–732
