# Supplementary material for: Next generation fatigue crack growth experiments of aerospace materials
Source: Sci Rep. 2024 Jun 18;14:14075. doi: 10.1038/s41598-024-63915-x (PMC11189514; doi:10.1038/s41598-024-63915-x)
Supplement: Supplementary file 2 — Supplementary Information 2. [file 41598_2024_63915_MOESM2_ESM.pdf]

Supplementary material for article:

Next generation fatigue crack growth experiments of aerospace materials

Tobias STROHMANN[1], David MELCHING[1], Florian PAYSAN[1], Eric DIETRICH[1], Guillermo REQUENA[1, 2], Eric BREITBARTH[1]

[1] German Aerospace Center (DLR), Institute of Materials Research, Linder Hoehe, 51147 Cologne, Germany

[2] Metallic Structures and Materials Systems for Aerospace Engineering, RWTH Aachen University, Aachen 52062, Germany

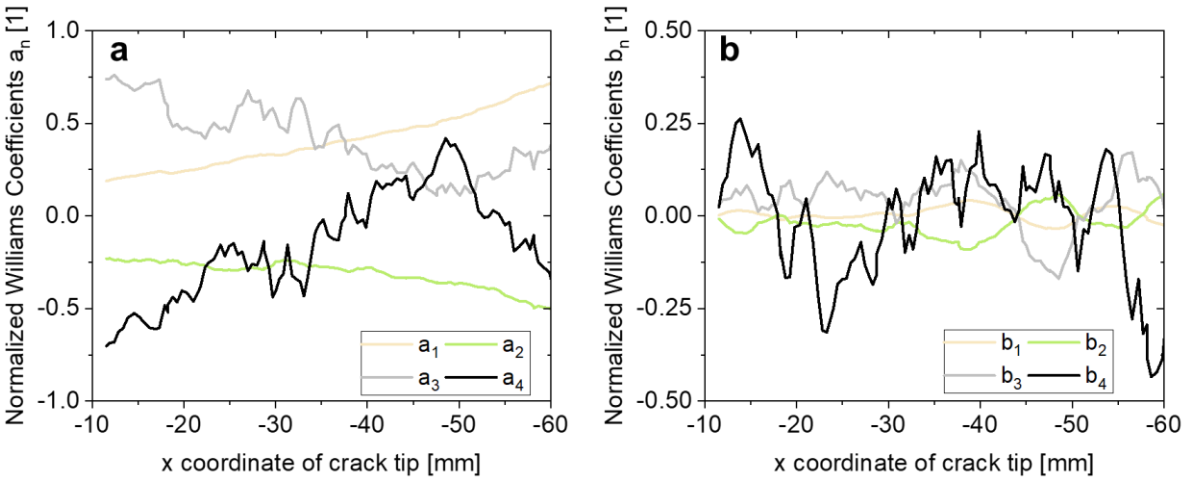

Supplementary Figure 1. Normalized Williams series coefficients a_1-a_4 and b_1-b_4 in **a** and **b**, respectively. The values are obtained by fitting the theoretical displacement field to the experimental DIC data using *CrackPy*. The individual coefficients highly differ with respect to their scale. To this purpose, all values were normalized with respect to their unit using eq. 1 and 2, respectively [1].

$$a_n = A_n \cdot \frac{1}{\frac{F}{Wt} \cdot W^{1-n/2}} \qquad \text{eq. 1}$$

$$b_n = B_n \cdot \frac{1}{\frac{F}{Wt} \cdot W^{1-n/2}} \qquad \text{eq. 2}$$

Supplementary Videos

Supplementary Video 1 – The video shows one loop of the automated robotic system upscaled to our biaxial test rig. The video starts with the crack growth phase for a given number of load cycles. This is followed by a 3D DIC calculation at maximum load. The calculated displacements are used to detect the crack tip positions of both crack tips (left and right). After the locations are transferred to the robot, the robot moves the microscope to the crack tips for local 2D HRDIC. Therefore, a region of 3×3 images is observed, locally. This is repeated for both sides of the crack and for maximum and minimum load. At the end of the loop, the robot moves back to its waiting position and the global DIC system captures another image at minimum load. The loop is shown as a time-laps.

Supplementary Video 2 – The video shows the crack tip coordinate as a function of load cycles for the data analysed in this work (uniaxial test rig, MT160 specimen). This is complemented by the displacement field at maximum and minimum load for the respective number of load cycles. At the bottom, three sub-regions of the sample are shown during crack propagation at a much higher resolution (HRDIC).

[1] B. L. Karihaloo, H. Abdalla, and Q. Z. Xiao. Coefficients of the crack tip asymptotic field for wedge splitting specimens. Eng Fract Mech, 70(17):2407–2420, 2003
